# Ubiquitination Events That Regulate Recombination of Immunoglobulin Loci Gene Segments

**DOI:** 10.3389/fimmu.2014.00100

**Published:** 2014-03-11

**Authors:** Jaime Chao, Gerson Rothschild, Uttiya Basu

**Affiliations:** ^1^Department of Microbiology and Immunology, College of Physicians and Surgeons, Columbia University, New York, NY, USA

**Keywords:** RAG proteins, AID, V(D)J recombination, class switch recombination, somatic hypermutation, ubiquitination, DNA repair

## Abstract

Programed DNA mutagenesis events in the immunoglobulin (Ig) loci of developing B cells utilize the common and conserved mechanism of protein ubiquitination for subsequent proteasomal degradation to generate the required antigen-receptor diversity. Recombinase proteins RAG1 and RAG2, necessary for V(D)J recombination, and activation-induced cytidine deaminase, an essential mutator protein for catalyzing class switch recombination and somatic hypermutation, are regulated by various ubiquitination events that affect protein stability and activity. Programed DNA breaks in the Ig loci can be identified by various components of DNA repair pathways, also regulated by protein ubiquitination. Errors in the ubiquitination pathways for any of the DNA double-strand break repair proteins can lead to inefficient recombination and repair events, resulting in a compromised adaptive immune system or development of cancer.

## Introduction

B cells are developmentally programed to undergo DNA double-strand breaks (DSBs) in the immunoglobulin (Ig) locus as they generate the antibody diversity required for adaptive immunity. Immature B cells reside in the bone marrow and undergo V(D)J recombination, using the DNA endonuclease activity of RAG1 and RAG2, to rearrange multiple gene segments and select a V(D)J exon in the immunoglobulin heavy chain (IgH) and a VJ exon in the immunoglobulin light chain (IgL) loci (1–3). Following V(D)J rearrangement, IgM^+^ B cells traverse to secondary organs (e.g., Peyer’s patches or the spleen) to undergo two additional DNA alteration events, namely class switch recombination (CSR) and somatic hypermutation (SHM), using the activity of the DNA cytidine deaminase activation-induced cytidine deaminase (AID) (4). CSR is a chromosomal rearrangement–deletion event requiring single-strand breaks in close proximity to each other on both DNA strands for selection of the particular antibody isotype the B cell will produce (5). SHM is the incorporation of point mutations in the recombined V(D)J exon of the IgH and IgL loci to increase the affinity of the expressed antibody for its cognate antigen (6). In this review, we discuss how DNA mutagenesis during V(D)J recombination, CSR, and SHM is regulated by various protein ubiquitination events.

Ubiquitination is a post-translation modification utilized as a regulatory mechanism by cells. The process involves the sequential actions of an ubiquitin-activating enzyme (E1), an ubiquitin-conjugating enzyme (E2), and an ubiquitin ligase (E3) to covalently attach ubiquitin to a lysine residue of the target protein. Deemed initially to be merely a system to mark proteins for degradation by the 26S proteasome, the diversity of ubiquitination and downstream events has recently been increasingly appreciated, reinforcing the possibility of an “ubiquitin code” as another layer in the multifaceted landscape of molecular regulation [reviewed in Ref. (7)].

## RAG Proteins and V(D)J Recombination

V(D)J recombination occurs in the IgH and IgL (comprised of Igκ and Igλ) chains of B cells. At the IgH locus, V(D)J recombination first connects a diversity (D) to a joining (J) segment to form a coding and a signal joint, followed by a second recombination event to bring together a variable (V) segment to the preformed DJ segment. IgL loci, on the other hand, do not contain D segments and are therefore subject to only one recombination event to form a VJ coding segment. For T cells, β and δ TCR loci parallel the IgH locus, first joining DJ segments before recombining to a V segment; α and γ TCR are analogous to IgL loci (1–3).

Recombination activating genes 1 and 2, encoding proteins RAG1 and RAG2 respectively, are necessary and sufficient for the breaks and rearrangements during V(D)J recombination. RAG1 and RAG2, collectively referred to as RAG in this review, are lymphoid-specific proteins that cleave and join DNA segments during V(D)J recombination [reviewed in Ref. (8)]. The RAG proteins specifically recognize recombination signal sequences (RSSs) that flank each V, D, and J segment. RSSs are composed of conserved heptamer and nonamer sequences separated by a non-conserved gap of either 12 or 23 base pairs, named 12RSS and 23RSS, respectively. Upon recognition and binding of a 12RSS or 23RSS, the RAG proteins form a complex that then captures the alternate 12RSS/RAG or 23RSS/RAG complex for synapsis to form a paired complex [reviewed in Ref. (9)]. Synapsis occurs exclusively between different RSS for efficient recombination, known as the “12–23 rule.” After the paired complex is formed, the RAG proteins nick and cleave 5′ of the RSS to produce a hairpin-closed coding end and a blunt signal end. Subsequent processing by non-homologous end joining (NHEJ) factors results in the final coding and signal joints, completing the recombination of a D to a J segment, a V to a DJ, or a V to a J segment. Signal joints containing the RSSs are excised, leaving coding joints as the operative DNA in the cell. How RAG1 and RAG2 interact with each other and in what exact sequence remains to be determined. However, the inherent ability of RAG to cause DNA breaks and recombination is of concern, particularly if lesions occur outside of warranted loci, namely IgH, IgL, or the TCR, engendering the possibility of translocations with oncogenes and/or transformation of the cell to a malignant state. Therefore, effective regulation of RAG1 and RAG2 is crucial both for efficient V(D)J recombination to generate the diversity in the adaptive immune system and for avoidance of genomic instability.

### RAG1 as an E3-ubiquitin ligase

RAG1 is the known catalytic component of the RAG complex, responsible for DNA binding and cleavage during V(D)J recombination [reviewed in Ref. (9)]. Core RAG1 is defined to include all necessary regions required for V(D)J recombination activity. Briefly, a well-defined nonamer-binding domain binds the nonamer sequence of the RSS, as the name implies. A central region (amino acids 528–760) includes a heptamer-binding domain and RAG2 interacting region, which is thought to involve the zinc finger region B (ZnB). Finally, three amino acids (D600, D708, and E962), known as the DDE motif, are important for DNA cleavage. Recently, interest in the significance of the non-core regions of RAG1 has increased, especially with the observation of the conserved N-terminal residues. Utilizing extrachromosomal recombination substrates and deletion and mutation analysis, the need for non-core RAG1 to enhance V(D)J recombination and fidelity has been suggested (10). The ZnA region of RAG1 includes an N-terminal RING domain that acts as an E3-ubiquitin ligase, with the potential to ubiquitinate a panel of targets for various downstream events (11–17), as well as the area which interacts with histone 3 (see below). The ZnA region is able to homodimerize, which may be significant for the E3-ubiquitin ligase activity of RAG1 and/or its regulation (14).

Through an *in vitro* experiment, the N-terminal RING domain of RAG1 has been shown to be capable of and necessary for mono-ubiquitinating test substrate S-protein in the presence of E2 enzymes UbcH10 or UbcH4 (11–17). Point mutations within the RING region, as well as deletion of half of the RING domain, strongly reduce the ubiquitination activity of the wild-type (WT) construct. Poly-ubiquitination was also observed in the presence of E2 UbcH5b, but in the absence of S-protein, suggesting that RAG1 has an auto-ubiquitination capacity. Because the RING domain of RAG1 is dispensable for V(D)J recombination activity, it is possible to conclude that RAG1 has an alternate enzymatic activity, though likely indirectly involved in V(D)J recombination. As noted above, RAG1 was identified as a potential E3-ubiquitin ligase utilizing a synthetic assay. Subsequent studies examining the RAG1 RING domain both *in vitro* and *in vivo*, however, were able to identify specific target substrates ubiquitinated by RAG1, and the nature of these ubiquitinations (11–16). It appears that the RAG1 RING domain is capable of interacting with multiple E2 ubiquitin-conjugating enzymes, with the specific E2 interaction possibly determining the subsequent ubiquitinated substrate(s). Accordingly, possible regulatory mechanisms for RAG1 and RAG2 activity and protein stabilization, as well as a range of alternative downstream pathways, have tentatively been identified.

**Figure 1:**
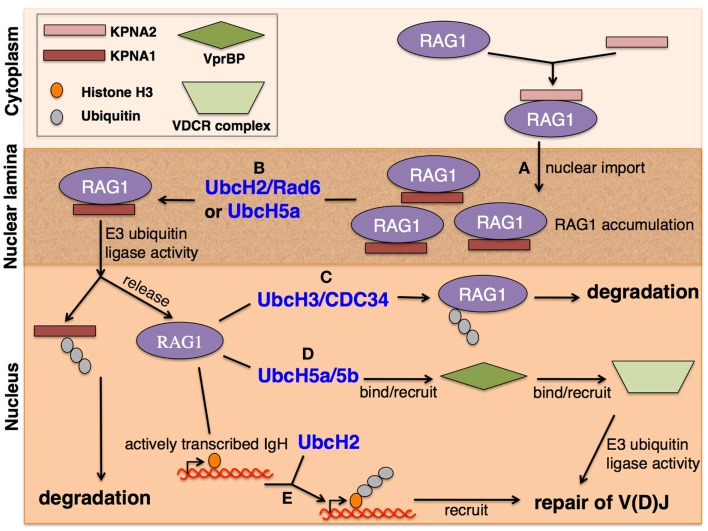
**RAG1 localization for V(D)J recombination**. **(A)** RAG1 is imported to the nucleus through nuclear pores upon KPNA2 binding to RAG1 NLS signal, located in the core region. RAG1 is thought to be retained within the nuclear lamina by binding KPNA1 upstream of the RING domain. RAG1 accumulation in the nuclear lamina promotes RAG1-dependent ubiquitination of KPNA1 in the presence of **(B)** E2 UbcH2/Rad6 or UbcH5a, allowing simultaneous degradation of KPNA1 by the 26S proteasome and release of RAG1 into the nucleus. RAG1 is capable of E3-ubiquitin ligase activity through its RING domain in the presence of a panel of E2 ubiquitin-conjugating enzymes. **(C)** Whereas E2 UbcH3/CDC34 promotes auto-ubiquitination of RAG1 for protein degradation, interaction with **(D)** E2 UbcH5a/5b enables RAG1 to bind with VprBP, which then binds to the VDCR complex to recruit its E3 activity, possibly to repair DNA breaks caused during V(D)J recombination. **(E)** E2 UbcH2 allows RAG1 to recognize actively transcribed DNA that was subject to DNA cleavage by core RAG1 by binding and ubiquitinating histone H3, specifically acetyl-H3.3 S31p, thereby tagging the DNA region for repair by NHEJ factors.

Ubiquitinated proteins are most often recognized and degraded by the 26S proteasome. However, RAG1’s unique RING domain structure, coordinating three Zn ions as opposed to the standard two of canonical RING domains, along with its ability to interact with a panel of E2 enzymes, suggest functions either alternate to or in addition to protein degradation (14). These alternative functions possibly are indirectly involved in V(D)J recombination and/or RAG regulation. RAG1 can be ubiquitinated in intact cells for subsequent degradation by the 26S proteasome, as evidenced by ubiquitinated species only being observed in a state of proteasome inhibition (11). Additionally, through an *in vitro* ubiquitination and pull-down assay, the RAG1 N-terminal region, containing the RING domain, Zn-binding domain, and basic upstream region, was observed to undergo auto-ubiquitination, specifically in the presence of E2 UbcH3/CDC34 (Figure 1C). In an experiment utilizing CH_3_-ubiquitin, which is unable to form poly-ubiquitin chains, it was shown that auto-ubiquitination of RAG1 primarily occurs at one conserved lysine residue, K233. Poly-ubiquitination was observed with WT ubiquitin, but does not necessarily depend on K48-linkage as poly-ubiquitin chains were seen with a K48R ubiquitin mutant. Finally, core RAG1 is more active than full-length protein *in vitro*, suggesting the importance of RAG1 auto-ubiquitination to potentially modulate its own turnover (11). With the RING domain and its E3-ubiquitin ligase activity missing, auto-ubiquitination of RAG1 cannot occur which may lead to the observed heightened activity of core RAG1, and possible promiscuous over-activity.

Beyond auto-ubiquitination and subsequent degradation, the RAG1 RING domain can interact with other E2 enzymes to ubiquitinate substrates involved in V(D)J recombination. One such substrate is histone H3 (13, 15). It was shown that the N-terminal region of RAG1 directly interacts with histone H3 and an intact RING domain is required for mono-ubiquitination of histone H3 *in vitro* and *in vivo* (13). Through experiments using both mutated and truncated proteins, it was determined that the N-terminal domains of both RAG1 and endogenous histone H3 directly interact. Structural analysis reveals RING mutations do not affect overall protein folding, just RAG1 activity. Furthermore, extrachromosomal V(D)J recombination assays demonstrate that point mutations within the RING domain of RAG1 cause deficient DNA joining, but not cleavage, at the endogenous IgH locus *in vivo*. It is interesting to note that patients with Omenn syndrome, a condition that results in combined immune deficiencies including reduced efficiency of V(D)J recombination, also harbor point mutations in the RAG1 RING domain (18, 19). Though inefficient V(D)J recombination from a RING domain mutation may be a readout of disrupted regulation from RAG1 auto-ubiquitination, ubiquitination of histone H3 may be an important factor during V(D)J recombination to (a) tag the RSS breaks to recruit DNA repair proteins, (b) destabilize the nucleosome or remodel the chromatin for DNA accessibility for repair, (c) promote RAG complex eviction to allow joining machinery to complete coding and signal joints, or (d) promote cell-cycle arrest (13). These possibilities are not mutually exclusive, and all have the potential to provide alternative pathways to mediate effective V(D)J recombination. Rather than one exclusive mechanism, it is likely a combination of the proposed models that is responsible for proper V(D)J recombination.

Subsequent investigations describe histone variant H3.3 as the target of RAG1 RING ubiquitination (15). Mutant RAG1 protein experiments and *in vitro* assays indicate binding between the RAG1 RING domain and the N-terminus of histone H3.3. From mass spectrometric analysis of H3 modifications, it is thought that acetylation and phosphorylation of H3.3 (acetyl-H3.3 S31p) are a mark of active chromosomes in mitotic cells, activate the histone as a substrate for RAG1 RING-dependent ubiquitination, and are upregulated during V(D)J recombination. Accordingly, acetyl-H3.3 S31p could act as a tag for recombining loci catalyzed by core RAG1 during V(D)J recombination (Figure 1E). Additionally, H3.3 was shown to have multiple sites of mono-ubiquitination in the presence of E2 UbcH2. It is possible that after DNA cleavage during V(D)J recombination, the RAG1 RING domain binds acetyl-H3.3 S31p to target the break site and recruit DNA repair complexes to complete the joining step and ensure faithful V(D)J recombination. RAG1 was also identified as an E3-ubiquitin ligase complex member, supporting the potential role of ubiquitinated histones as marking breaks to recruit repair proteins (12). Evidence for this role includes the observation that full length, but not core, RAG1 with core or full-length RAG2 co-purifies with a complex containing VprBP, DDB1, Cul4A, and Roc1 (VDCR complex) *in vitro*, which is known to act as a recruitment scaffold for repair proteins (Figure 1E).

Although there are multiple reports of RAG1 being capable of E3-ubiquitin ligase activity, there are discrepancies as to with which E2 enzyme the RAG1 RING domain interacts. Since the E3 enzyme of most ubiquitin reactions largely determines the specific substrate to be ubiquitinated, it is interesting to note that it is the E2 enzyme instead that appears to target the downstream ubiquitinated product in the context of E3 RAG1 (Figure 1). In previously described studies, the presence of different E2 enzymes results in different substrates of RAG1 RING. This observation could be a consequence of differing experimental designs and the lack of a reliable *in vivo* system. Alternatively, the results could properly depict the broad range of potential proteins subject to ubiquitination by the RAG1 RING E3 ligase activity for purposes other than protein degradation.

As a potential model, upon RAG1 nuclear import (Figure 1A), the interaction and ubiquitination of KPNA1 releases RAG1 from the nuclear lamina with the assistance of E2 UbcH2/Rad6 or UbcH5a (20) (Figure 1B). The freed nuclear RAG1 is then able to auto-ubiquitinate in the presence of E2 UbcH3/CDC34, regulating its own protein levels by marking itself for degradation (11) (Figure 1C). Alternatively, RAG1 can act with E2 UbcH2 to target and ubiquitinate histone H3 of actively transcribing DNA for both processing by V(D)J recombination and for tagging the cleaved DNA to recruit proteins involved in DNA repair to promote robust and efficient NHEJ (13) (Figure 1E). Simultaneously, RAG1 with E2 UbcH5a/5b can interact with VprBP of the VDCR complex, an E3-ubiquitin ligase complex, to recruit repair proteins during V(D)J recombination (12) (Figure 1D). The proposed model presents another level for the cell to regulate RAG1 by regulating E2 enzymes available for use. For example, if E2 UbcH2/Rad6 and UbcH5a are not available during S phase of the cell cycle, RAG1 would not be able to ubiquitinate KPNA1 and therefore unable to release itself from the nuclear lamina to perform its catalytic role. Regulation of these E2 enzymes prevents promiscuous RAG1 activity outside of appropriate V(D)J recombination events. Similarly, the presence of E2 UbcH3/CDC34 or UbcH2 are important in regulating RAG1 protein stabilization or recruiting repair proteins for efficient V(D)J recombination, respectively. The second activity of RAG1 as an E3-ubiquitin ligase, in addition to catalyzing DNA breaks, provides alternative mechanisms in modulating V(D)J recombination that include multiple pathways for RAG1 activity regulation.

### Regulation of RAG2 by ubiquitination

**Figure 2:**
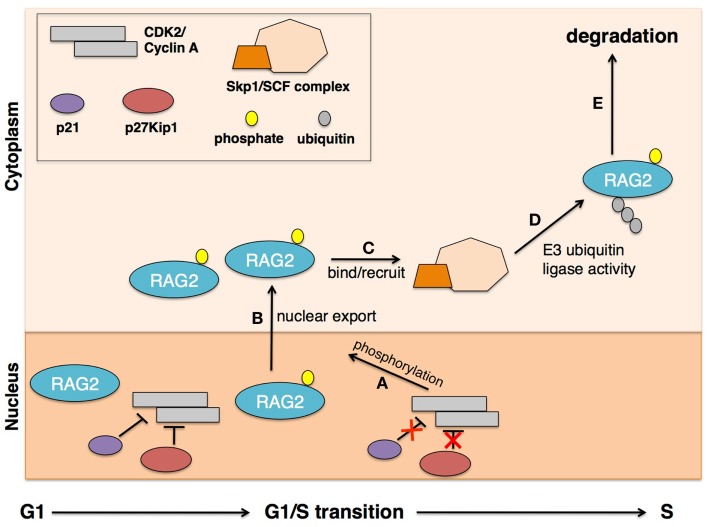
**Cell-cycle-dependent RAG2 localization for V(D)J recombination**. Periodic RAG2 accumulation is dependent on cyclin A/CKD2 activity. In the G1 cell-cycle phase, cyclin A/CKD2 is inhibited by p27Kip1 and p21, and RAG2 protein is stable and abundant in the nucleus. Upon transition into S phase, p27Kip1 and p21 are degraded, allowing **(A)** cyclin A/CKD2 activity to phosphorylate RAG2 at Thr-490 residue. This phosphorylation event triggers **(B)** nuclear export of RAG2 where it can **(C)** bind Skp2 in the cytoplasm to recruit the SCF complex. The SCF complex is a known E3-ubiquitin ligase complex that **(D)** ubiquitinates RAG2 for **(E)** rapid degradation by the 26S proteasome. This model explains the observed periodic accumulation of RAG2 during G1, coinciding proper cell-cycle activity for efficient V(D)J recombination.

Though the function remains rather elusive, RAG2, like RAG1, has a defined core region (amino acids 1–383) located at the N-terminal end and with core RAG1, is required for V(D)J recombination [reviewed in Ref. (17)]. The RAG2 core is known to be critical for DNA cleavage and enhances DNA binding and specificity of the RAG complex, probably through its interaction with RAG1, as RAG2 itself has little or no DNA-binding activity. The RAG2 non-core region includes a PHD domain that specifically binds H3K4me3 to guide RAG2 to active chromatin and enhances the catalytic activity of the RAG complex. An important characteristic of RAG2 is its periodic accumulation and degradation in relation to the cell cycle (21). RAG2 is phosphorylated at residue Thr-490, located in the C-terminal non-core region, by the cell-cycle-dependent protein cyclin A/CDK2. Cyclin A/CDK2 is upregulated at the G1/S phase transition and maintained through entry into M phase. It is known that p27Kip1, a cyclin-dependent kinase inhibitor, blocks the activity of cyclin A/CKD2, in effect stabilizing nuclear RAG2 protein levels (21, 22). It is therefore hypothesized that at the G1/S phase transition, p27Kip1 is degraded to allow phosphorylation of RAG2 at Thr-490 by cyclin A/CDK2, leading to RAG2 protein degradation and thereby regulating RAG2 protein levels in a cell-cycle-dependent manner (Figure 2A).

Specifically, it was shown that RAG2 degradation is mediated by ubiquitination and subsequent activity of the 26S proteasome, as poly-ubiquitinated RAG2 species were observed upon treatment with a specific 26S proteasome inhibitor (23). Furthermore, ubiquitination assays comparing full length, T490A mutant, and C-terminal-deleted RAG2, with and without 26S proteasome inhibition, suggest that sites for ubiquitin ligase interaction and ubiquitination are located at the N-terminal region of RAG2. Though the C-terminal region was observed to be inhibitory to ubiquitination and degradation of RAG2, phosphorylation of the Thr-490 residue abrogates this C-terminal inhibitory activity. These findings demonstrate self-regulatory functions of RAG2 through interactions of different regions of the protein. As alluded to earlier, it was observed that RAG2 localizes in both the cytoplasm and the nucleus, and this subcellular localization is cell-cycle dependent. Western blot analysis shows cytoplasmic RAG2 is less stable than nuclear RAG2 (23). Along with active p27Kip1, the C-terminal region helps to retain RAG2 within the nucleus. However, post-translational phosphorylation of Thr-490 of RAG2 seems to abrogate the C-terminal inhibitory function, allowing nuclear RAG2 export into the cytoplasm (Figure 2B).

Cytoplasmic localization renders RAG2 accessible for ubiquitination and degradation. It was observed that phosphorylated Thr-490 of RAG2 is an interacting site for another cell-cycle regulator, Skp2-SCF (24). Via biochemical fractionation, E2 Cdc34, commonly associated with the SCF family of E3-ubiquitin ligases, was identified as stimulating RAG2 ubiquitination. Further biochemical assays determined Skp2-SCF as the specific E3-ubiquitin ligase complex for RAG2 ubiquitination, with confirmation from subsequent *in vivo* mutation and knock-down experiments. G1 and S/G2/M phase cells collected from Skp2-deficient mice demonstrated that the periodic accumulation of RAG2 is dependent on Skp2, as RAG2 protein levels from S/G2/M phase were elevated in Skp2-deficient mice. Other *in vitro* experiments confirmed the dependence on the Skp2-SCF complex in regulating RAG2 protein with the cell cycle. Skp2 was also shown to bind Skp1 of the SCF complex, which recruits the complex’s E3 activity to RAG2 for ubiquitination and degradation. The SCF complex possesses alternative roles for targeting other cell-cycle factors such as p27Kip1 and p21 for degradation (Figures 2B,C). Interestingly, both p27Kip1 and p21 are inhibitors of cyclin A/CDK2 and are therefore negative regulators of RAG2 degradation. The importance of SCF E3-ubiquitin ligase activity is highlighted by the range of target proteins, which all ultimately converge to regulate the activity of RAG2 and permit proper V(D)J recombination.

The findings from experiments investigating RAG2 activity and degradation have emphasized RAG2’s role in regulating V(D)J recombination activity to appropriate phases of the cell cycle. DNA breaks during S phase are potentially harmful for cells as they can lead to translocations and lymphomas when misrepaired by homologous recombination (HR). It is therefore crucial to limit V(D)J recombination activity within the G1 phase; the restriction appears to be controlled by RAG2 localization and stabilization. RAG2 nuclear accumulation is only observed during G1, presumably because upon transition to S phase, p27Kip1 is degraded, relieving suppression of cyclin A/CDK2 activity and permitting phosphorylation of RAG2 at Thr-490 (21, 22). The phosphorylation at Thr-490 allows for nuclear export where the phosphorylated residue interacts with Skp2 to recruit the E3-ubiquitin ligase activity of the SCF complex (24). Ubiquitination of RAG2 allows for rapid degradation of the protein upon entering S phase, thereby halting any potential off-target activities of RAG (Figure 2).

The periodic accumulation and degradation of RAG2 has significant implications for V(D)J recombination activity. Since RAG2 together with RAG1 is required for catalysis of V(D)J recombination events, its nuclear retention during G1 is important. However, upon S phase entry, RAG2 is rapidly degraded to prevent non-specific DNA cleavage events. Without RAG2, RAG1 can remain bound to DNA where its RING E3-ubiquitin ligase activity can ubiquitinate histone H3 and/or VprBP, as discussed earlier. Though RAG1 is catalytically compromised in the absence of RAG2 for V(D)J recombination activity, it still retains its E3-ubiquitin ligase activity. This proves significant as ubiquitination of H3.3 possibly tags DNA for repair while ubiquitination of VprBP is thought to recruit the E3 activity of the VDCR complex. Together, degradation of RAG2 during the G1/S phase transition simultaneously halts V(D)J recombinase activity and recruits NHEJ repair proteins to sites of DNA breaks for efficient and productive V(D)J recombination.

## AID-Mediated Class Switch Recombination and Somatic Hypermutation

Upon completion of V(D)J recombination in the bone marrow, immature IgM^+^ B cells migrate to secondary lymphoid tissues for further DNA alterations events, namely CSR and SHM, which are mediated by AID. Following antigen-dependent activation, germinal center B-lymphocytes undergo CSR to generate antibodies with different effector functions, and SHM to increase the affinity of the antibody for its cognate epitopes, a process also known as affinity maturation (6, 25, 26). The IgH locus has multiple constant region genes (C_H_) that are preceded by G-rich switch sequences (S) and subject to CSR. In mice, there are eight sets of C_H_ exons organized as 5′–V(D)J–Cμ–Cδ–Cγ3–Cγ1–Cγ2b–Cγ2a–Cε–Cα–3′, and each S sequence has its own cognate promoter that is influenced by enhancer elements, such as the 3′ regulatory region enhancer (3′ RR) (5). This allows for transcription of stimulated B cells at Sμ and another S sequence, such as Sγ1, Sγ3, Sε, or Sα (Cδ lacks an upstream switch sequence), resulting in selection of a constant region that now encodes IgG1, IgG3, IgE, or IgA, respectively. Transcription-dependent generation of a DNA DSB mediated by AID at the donor Sμ sequence and downstream acceptor switch sequence leads to a recombination–deletion event that removes the intermediate sequences and propagates both S_donor_–S_acceptor_ synapsis and catalysis of CSR [reviewed in Ref. (1, 4)]. Additionally, B cells carry out SHM by introducing mutations, also catalyzed by AID, in the variable regions of the IgH and IgL loci, which, when synthesized, are in physical contact with the antigen during an immune challenge. The mutations in these segments occur at a frequency much higher than in other regions of the genome. The mutations are initiated at RGYW motifs by AID and spread as a consequence of downstream events orchestrated by the mismatch repair (MMR) and base excision repair (BER) pathways (27, 28) [reviewed in Ref. (6, 29, 30)]. Understanding the molecular mechanism by which transcription within various regions of the Ig locus coordinates with the mutagenic activity of AID to generate and regulate programed DNA lesions has been a challenge. Comprehension is, however, necessary to fully understand AID and its tumorigenic potential upon misregulation.

### Activation-induced cytidine deaminase and protein (in)stability through ubiquitination

Activation-induced cytidine deaminase is a single-strand cytidine deaminase that utilizes transcription-dependent mechanisms to generate single-strand DNA (ssDNA) structures that allow mutagenesis of target DNA substrates of B cells (31). Chromatin immunoprecipitation (ChIP) of AID from CSR-stimulated B cells followed by high throughput sequencing of AID-associated DNA fragments (ChIP-seq) reveals that AID can bind various regions of the B-cell genome, inside and outside the Ig loci, presenting opportunities for genomic instability (32). Therefore, understanding the physiological pattern and distribution of AID-generated mutations at AID’s target sequences is vital. Changes in AID mutation distributions at S regions can decrease CSR efficiency and generate DSB intermediates causing oncogenic IgH translocations while similar aberrant mutation patterns at the variable regions during SHM can alter antibody specificity for antigen (6, 33–36). Indeed, post-translation modifications and co-factors of AID have been identified and proposed to affect its activity through multiple mechanisms, such as (a) stimulation of AID’s DNA deamination activity, (b) linking AID to the Ig transcription machinery, (c) establishing a physiological DNA deamination pattern on both strands of DNA, and (d) linking AID to the downstream DNA repair machinery [reviewed in Ref. (4)]. Two separate studies investigated the regulatory role of post-translation AID ubiquitination during CSR and SHM (37, 38). Unlike RAG2, AID protein stability is not associated with phases of the cell cycle, but rather with subcellular localization. Utilizing expression constructs, AID mutants, and pulse-chase experiments, the Reynaud group showed that in mouse B cells and 293T cells, nuclear AID is subject to rapid turnover upon poly-ubiquitination (37). Nuclear-restricted AID was shown to have enhanced mutagenic activity in both Ig and non-Ig loci, demonstrating the importance of controlling AID off-target activity. As no specific lysine residue was determined to be the target of ubiquitination, it remains unclear whether several sites are poly-ubiquitinated or if N-terminal residues of AID are targeted. This work highlights the multilayer regulation of AID function, including protein stability and turnover (37).

Since AID is known to promote oncogenic mutations in the B-cell genome (4, 39, 40) and its protein levels are likely controlled by ubiquitin-mediated degradation (37), it is crucial to identify mechanisms of AID ubiquitination, specifically in the context of the transcription complex. To this end, the Papavasiliou group recently discovered that the RING E3 ligase RNF126, with E2 UbcH5b, can mono-ubiquitinate AID in cell-free assay conditions and in 293T cells (38). Though the functional implications of RNF126-mediated AID ubiquitination were not investigated, the authors provide several mechanistic insights concerning the potential role of AID ubiquitination. Auto-ubiquitination and ubiquitin binding, due to the presence of an N-terminal ubiquitin-binding domain of RNF126, may prevent recruitment of PCNA and translesion polymerase, which inhibits spreading of AID-generated mutations. Alternatively, RNF126 could be involved in regulating transcription initiation at promoters of AID target genes, presenting important implications for AID targeting, discussed below. Better understanding of RNF126 in AID regulation will be dependent upon generation of RNF126-deficient mouse models (38).

### Ubiquitination of AID-associated RNA polymerase II

Beyond post-translation modifications to regulate protein stability, co-factors are also important for proper AID activity. AID is proposed to bind the paused and/or stalled state of RNA polymerase II (RNA pol II), consistent with its transcription-dependent activity (4, 39, 40). Whereas “paused RNA pol II” is bound to DNA and is positioned at DNA sequences proximal to genic transcription start sites (TSSs) prior to entering transcriptional elongation (41), “stalled RNA pol II” is positioned on template DNA during RNA pol II elongation (42). RNA exosome and Spt5 are co-factors of both RNA pol II and AID in B cells (43, 44). Though the 3′ → 5′RNA exonuclease complex RNA exosome is predominantly associated with the stalled RNA pol II complex, Spt5 is associated with both paused and stalled RNA pol II complexes (42). ChIP-seq data show AID-bound sequences have high occupancy by RNA pol II molecules that are either in the elongation phase or in the paused or stalled state (43). Consistent with these observations, RNA pol II is enriched at various regions of IgS sequences, an AID target (45). The switch sequences are enriched with AID and Spt5 (32, 43). These results present a role for the transcription machinery in AID targeting and the importance of regulating the transcription complex to prevent promiscuous activity of AID. Below, we will discuss the role of ubiquitination in regulating these processes.

Many AID-induced mutations occurring during CSR (36) or SHM (46) are significantly downstream from the transcription start sites of V genes or switch sequences (as opposed to 100 bps downstream of TSS where RNA pol II promoter-proximal pausing occurs). This downstream localization is also true for other AID target genes (47). Thus, it is likely that elongation-stalled RNA pol II is an adequate complex for recruitment of AID during SHM and CSR. RNA exosome recruitment to stalled RNA pol II requires the presence of a nascent RNA with a free 3′-end. Stalled RNA pol II complexes occur during encounter with various obstacles caused by (a) the presence of antisense transcription, (b) secondary DNA structures including those caused by G-richness on the non-template strand of S sequences, (c) variation in the levels of elongation promoting chromatin modifications, and (d) the presence of mutations or DNA lesions on the template DNA (4, 39, 40). Resolution of the stalled complex includes mechanisms of backtracking or early termination of the complex, which dissociates the RNA from RNA pol II (41), or ubiquitin-mediated destabilization of RNA pol II (42, 48). These mechanisms reveal a free 3′-end RNA substrate for RNA exosome, a co-factor of AID.

**Figure 3:**
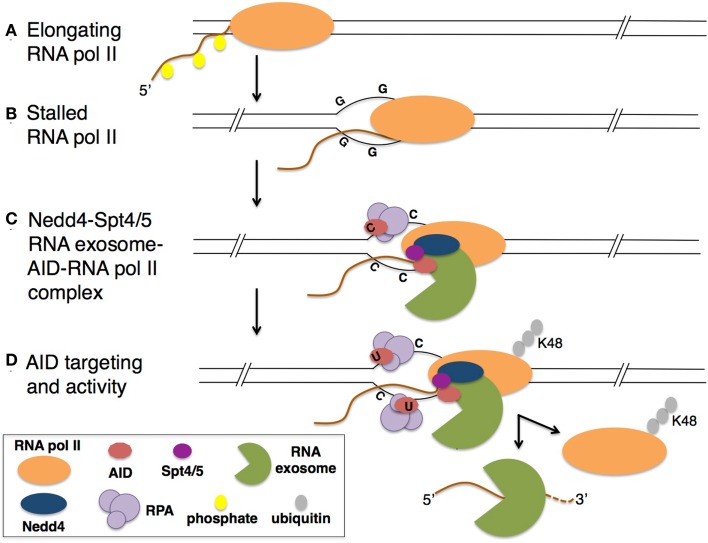
**Model for E3 ligase Nedd4 in mediating CSR**. **(A)** During transcription of the B-cell genome, RNA pol II in the elongation complex encounters **(B)** a transcriptional block, such as secondary structures formed in the G-rich switch regions of Ig genes. **(C)** Stalled RNA pol II exists in a complex with interacting E3 ligase Nedd4, Spt4/5, RNA exosome, and AID. RPA-associated AID targets the non-template DNA strand. **(D)** K48-poly-ubiquitinated RNA pol II is released, relieving the 3′ end of the RNA transcript available for RNA exosome degradation. The release of the DNA/RNA hybrid allows AID to target both template and non-template ssDNA (73).

Recently, it was shown that E3-ubiquitin ligase Nedd4 identifies and ubiquitinates AID-associated RNA pol II (49). Through immunoprecipitation experiments, AID was shown to interact with RNA pol II, stalling factor Spt5, and RNA exosome. AID-associated RNA pol II is poly-ubiquitinated in a Nedd4-dependent fashion. B cells obtained from Nedd4-mutant mice demonstrate increased stability of IgH germline transcripts that are expressed from DNA switch sequences subject to AID mutations, indicating that Nedd4 activity promotes processing of germline transcripts, possibly by recruiting AID. Finally, B cells from Nedd4-mutant mice are impaired in CSR and have a compromised mutation rate in the 5′ Sμ sequence, an AID target. These observations highlight the requirement of Nedd4 and its ubiquitin ligase activity for proper AID-associated RNA pol II activity during antibody diversification mechanisms (Figure 3).

The requirement of AID-associated RNA pol II ubiquitination has implications for AID targeting. First, ubiquitin-mediated RNA pol II destabilization exposes the 3′ end of the RNA pol II-associated nascent transcript for degradation by RNA exosome. Nedd4 activity therefore could contribute toward the generation of ssDNA substrates on both the template and non-template strands of DNA for AID deamination by triggering RNA exosome activity (Figure 3), possibly as an alternative mechanism to RNA pol II backtracking. In addition to facilitating targeting of AID activity to specific regions of the genome, Nedd4 may also prevent accumulation of stalled RNA pol II at AID target regions in the B-cell genome to prevent generation of aberrant DNA DSBs. Collision of the stalled RNA pol II with replication machinery may be responsible for DNA DSBs and chromosomal translocations. Whether Nedd4 ubiquitination activity is required for RNA pol II destabilization during SHM to promote mutations in variable region genes or at oncogenic targets of AID is important to determine.

In addition to direct ubiquitination of AID to regulate its nuclear localization and stability, ubiquitination also appears to indirectly control AID targeting to its physiological DNA substrates by facilitating AID-associated RNA pol II destabilization to reveal substrates for RNA exosome, an AID co-factor. Protein stability and specific targeting of nuclear AID are vital for effective CSR and SHM, and therefore regulation of both processes is required to prevent off-target activity and genomic instability.

## Repair of DNA Double-Strand Breaks During B-Cell Development

As immature B cells are subject to programed DNA DSBs during V(D)J recombination, CSR, and SHM during development, appropriate repair of these DSBs is essential for proper B-cell maturation and prevention of disease and oncogenesis. Importantly, both RAG- and AID-induced DSBs depend on NHEJ, not HR, for repair (1, 17). DSBs are initially recognized by the Ku70/Ku80 heterodimer to recruit kinase DNA-PK [reviewed in Ref. (50)]. Other DSB repair factors are then recruited, including XRCC4-ligase IV, Pol μ, Pol λ, and TdT in lymphocytes. Phosphorylated histone H2AX on Ser139 (γ H2AX) by DNA-PK is a well-known mark for DNA damage and recruits MDC1 (mediator of DNA damage checkpoint protein 1) via interaction with the BRCT motif (51). Upon interacting with phosphorylated ATM through its FHA domain, MDC1 both stabilizes the histone modification and amplifies the γ H2AX signal (52). ATM then phosphorylates TQXF motifs of MDC1. It is at this point in the NHEJ repair pathway that ubiquitination assumes its prominent role (53, 54). The initial recognition and recruitment of repair proteins to DSBs is heavily dependent on phosphorylation events (e.g., kinase activity and autophosphorylation of DNA-PK), but it is the downstream repair events that appear to be dependent on E3-ubiquitin ligases (55).

### Role of E3 ligases RNF8 and RNF168 during repair of DNA DSBs

At the crossroads between phosphorylation- and ubiquitin-dependent events in DSB repair, phosphorylated MDC1 is recognized by the E3 ligase RNF8 through its FHA (forkhead associated) domain (56). Two separate groups described RNF8 binding to MDC1 for ubiquitinating damaged-associated histones (57, 58). Mailand et al. used a bioinformatics approach to panel motifs of known DSB regulators to identify RNF8. Its function was tested and it was observed to colocalize with γ H2AX at DSBs; colocalization was abrogated in the absence of MDC1 (57). To further characterize the relationship between RNF8 and MDC1, Mailand used a combination of biochemical and real-time imaging techniques to reveal the direct interaction of RNF8 and MDC1, which requires the FHA domain and TQXF motif of RNF8 and MDC1, respectively. Furthermore, RNF8 was shown to rapidly accumulate at DSBs with the same kinetics as MDC1, preceding 53BP1 and BRCA1 accumulation, suggesting that RNF8 functions upstream of 53BP1 and BRCA1, factors involved with NHEJ and HR, respectively. The second group, Huen et al., used a tagged-RNF8 construct to biochemically determine its colocalization with γH2AX and other known damage response proteins, such as MDC1 and 53BP1, to DSBs (58). Consistent with the previously described study, RNF8 was shown to function downstream of γH2AX and MDC1 recruitment and to interact directly with phosphorylated TQXF motifs of MDC1 via its FHA domain. Both groups demonstrate the necessity of both the FHA and RING domains for complete RNF8 function. Importantly, the RING domain is capable of ubiquitin ligase activity on H2A (57) and H2AX (58) in *in vitro* ubiquitination assays. In the presence of E2 Ubc13, RNF8 is capable of mono- and di-ubiquitinating H2AX. Taken together, these findings present evidence for a functional link between RNF8 and H2A, possibly modifying histones to reveal buried substrates necessary for downstream interactions by NHEJ repair proteins, such as 53BP1.

Subsequent to the two previous studies, the Durocher group showed that RNF8 also impairs 53BP1 focus formation (53). Depleting RNF8 abrogated 53BP1 foci without disrupting MDC1 foci. Further investigation revealed that the N-terminal FHA domain and C-terminal RING domain of RNF8 are both necessary for 53BP1 focus formation as mutation in either domain abolishes 53BP1 focus formation. This study highlights the significance of γH2AX- and MDC1-dependent RNF8 response to DSBs in recruiting 53BP1 accumulation. However, direct interaction between phosphorylated MDC1 and 53BP1 was not observed. This is not unexpected, as 53BP1 is known to recognize H4K20me2, suggesting an additional mediator exists downstream of RNF8 to recruit 53BP1 (59).

**Figure 4:**
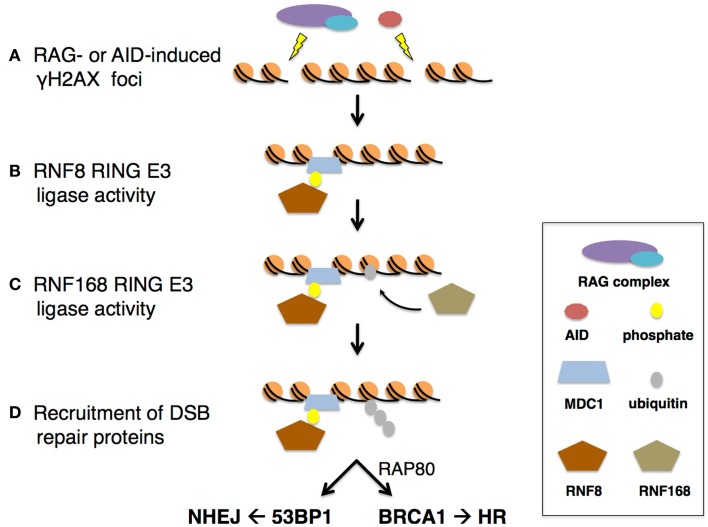
**RNF8- and RNF168-mediated repair of RAG- or AID-induced DSBs**. **(A)** DSBs from RAG or AID activity result in γH2AX foci formation to recruit MDC1. **(B)** RNF8 recognizes ATM-dependent phosphorylated MDC1 via its FHA domain. The RING domain of RNF8 then ubiquitinates DSB-associated chromatin. **(C)** RNF168 recognizes ubiquitin-modified breaks via its MIU motif. RNF168 is recruited to DSB sites and **(D)** extends the RNF8-mediated ubiquitin conjugates. The RNF8/RNF168-dependent poly-ubiquitinated products are then recognized for repair completion by BRCA1 through RAP80 for HR, or 53BP1 for NHEJ.

Two groups utilizing similar genome-wide siRNA screens identified RNF168 as the additional mediator for 53BP1 focus formation (60, 61). Both groups confirmed RNF168 acts downstream of RNF8 in the repair pathway, as depletion of MDC1 and RNF8 abrogated RNF168 foci, but the foci were unaffected by depletion of 53BP1 or BRCA1 (61). Furthermore, knock-down of RNF168 results in RNF8 accumulation at DNA damage sites but RNF8-dependent ubiquitinated chromatin is unstable, suggesting transient activity of RNF8 that is stabilized by RNF168 (60). As 53BP1 foci were dependent on both the N-terminal RING and two MIU (motif interacting with ubiquitin) domains of RNF168, the RING domain of RNF168 was then tested for ubiquitin ligase activity. In *in vitro* ubiquitin assays, RNF168 was indeed capable of E3 activity, with direct interaction with Ubc13, the only known E2 capable of catalyzing formation of K68-ubiquitin chains. Subsequent experiments confirmed RNF168 specifically ubiquitinates H2A type histones, including H2A and H2AX, with E2 Ubc13 to form K63-ubiquitin chains. The poly-K63-ubiquitinated histones were shown to be dependent on both RNF8 and RNF168. These findings suggest a model whereby RNF8 is first recruited to sites of DSBs via its FHA domain, recognizing phosphorylated TQXF motifs of MDC1 (Figure 4B). RNF8 is then able to initiate the ubiquitination of γH2AX to recruit RNF168 via its MIU domains to promote K63-ubiquitin chain extension (60) (Figure 4C). In this way RNF168 stabilizes and amplifies the transient ubiquitin conjugate signals from RNF8 for recruitment of downstream repair proteins (Figure 4D).

Though the sequential recruitment of RNF8 followed by RNF168 is thought to mimic their sequential activity at DSBs for repair (Figure 4), recent observations by Mattiroli et al. challenge this model. *In vitro* assays confirm the ability of RNF8 and RNF168 to ubiquitinate free histone variants (62). However, in the presence of purified nucleosomes, where the octamer surrounds DNA, only RNF168 is capable of ubiquitinating H2A. Upon further investigation, results suggest a mechanism whereby RNF168 recruitment to sites of DSBs is RNF8-dependent, but RNF8-mediated ubiquitin chain extension is dependent upon mono-ubiquitination by RNF168. If this revised model holds, it will be interesting to determine how RNF168 is initially recruited. Panier et al. suggest RNF168 recruitment involves two “waves” of RNF168 accumulation at DSBs, and describe the role of p97 in removing RNF8-mediated ubiquitin conjugates to unmask H4K20me2 for 53BP1 interaction and recruitment (63).

### RNF8- and RNF168-dependent recruitment of 53BP1 to DSBs

Though the RNF8- and RNF168-dependent recruitment of repair proteins to DSBs is well-documented, the mechanism by which 53BP1 recognizes its substrate, H4K20me2, remains unclear. As mentioned above, RNF168 was shown to preferentially produce K63-ubiquitin chains via its interaction with E2 Ubc13. How, then, is 53BP1 able to recognize RNF8- and RNF168-modified DSBs? Because of its effect on DNA repair and its important role in various ubiquitin-dependent processes in the cell, the ubiquitin-selective segregase p97 was investigated in relation to RNF8 and RNF168 (64, 65). The accumulation of p97, and its adaptor protein NPL4, at DSB sites was shown to be RNF8-dependent but RNF168-independent (66). Moreover, RNF8 RING domain and free nuclear ubiquitin must be present for p97 foci formation. *In vivo* co-affinity purification assays show p97 and RNF8 interact in a complex, with RNF8 directly binding ubiquitin and p97 interacting with ubiquitinated moieties (67). Real-time recruitment kinetics show p97 accumulation occurring after MDC1 but before 53BP1, supporting observations of MDC1- and RNF8-dependent recruitment for efficient downstream 53BP1 recruitment (66, 67). BRCA1 focus formation was not affected by p97 depletion, confirming the specificity of p97 for 53BP1 recruitment (66).

Though 53BP1 recruitment is mediated by RNF168 ubiquitin ligase activity, 53BP1 interacts with H4K20me2, not ubiquitinated moieties (59). p97 was also observed to bind to and regulate the turnover of RNF8-dependent K48-ubiquitin chains (67). This suggests a mechanism by which RNF8 catalyzes K48-ubiquitin chains to recruit p97 through its ubiquitin adaptor UFD1–NPL4. The K48-ubiquitin conjugates are then removed by the ATPase-driven segregation activity of p97, which causes a rearrangement of the DSB-associated chromatin complex, possibly revealing buried H4K20me2, allowing interaction with and recruitment of 53BP1 (67). This provides insight into the factors directly mediating 53BP1 focus formation downstream of RNF8 activity for proper repair by NHEJ. Importantly, the polycomb complex also interacts with H4K20me2 and with higher affinity than 53BP1 (61). However, upon DNA damage and subsequent γH2AX focus formation, L3MBTL1 (a polycomb complex protein) is poly-K48-ubiquitinated with subsequent increased p97 interaction and decreased H4K20me2 association (66). Taken together, it is possible that RNF8 poly-ubiquitinates the H4K20me2-associated polycomb complex at DSB-associated chromatin to recruit p97–UFD1–NPL4 through direct binding of ubiquitin moieties. The ATPase-driven segregation activity of p97 then mediates the turnover of K48-ubiquitinated polycomb complex, relieving H4K20me2 sites for recruitment of 53BP1. In this way, p97 is essential for connecting the E3-ubiquitin ligase activities of RNF8 to 53BP1 recruitment for NHEJ by revealing H4K20me2 binding sites for 53BP1.

### 53BP1 directs repair toward NHEJ and away from HR

In RNF168-deficient cells, 53BP1 foci are abolished and inefficient repair of DSBs during V(D)J recombination and CSR are observed, emphasizing the importance of RNF168 for 53BP1 recruitment and repair of RAG- and AID-induced DSBs. Since V(D)J recombination and CSR require repair of distal DNA breaks, the role of 53BP1 in promoting NHEJ has been investigated.

In 53BP1 knockout mice, long-range V(D)J recombination is impaired, but short-range recombination between D–J segments is not defective, supporting previous observations of increased frequency of short-range intra-switch recombination during CSR in the absence of 53BP1 (68). In addition to repair defects, 53BP1-deficient cells also exhibit end resection of unpaired V(D)J recombination-induced coding ends (69). Inhibiting DNA end resection is important for NHEJ-mediated repair of DSBs as the resulting ssDNA presents microhomologies for repair by alternative end joining (A-EJ) and HR pathways. Taken together, 53BP1 recruitment to DSB sites presents alternative, though not mutually exclusive, roles for repair during V(D)J recombination and CSR, as discussed below.

**Figure 5:**
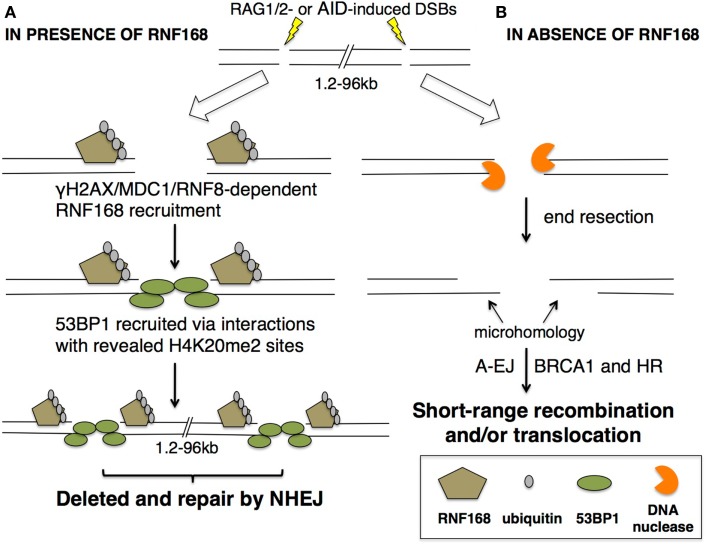
**53BP1 recruitment to DSBs is dependent on RNF168**. Under normal physiological conditions, DSBs occur as a consequence of RAG1/2 or AID activity. DSBs lead to recruitment of **(A)** RNF168 in an γH2AX/MDC1/RNF8-dependent manner. RNF168 accumulation and E3 ligase activity poly-ubiquitinates the DSB-associated chromatin, revealing buried H4K20me2 sites for 53BP1 interaction, possibly by rearranging chromatin structure. 53BP1 recruitment and oligomers protect open DNA ends and mediate long-range recombination and repair that occurs during V(D)J recombination and CSR. **(B)** In the absence of RNF168, 53BP1 foci do not occur, leaving DNA available for attack by nucleases for end resection. End resection can then present microhomologies, resulting in A-EJ or BRCA1-mediated HR. Both RNF168- and 53BP1-deficient cells result in short-range recombination, translocations, and defective V(D)J recombination and CSR. In this way, 53BP1 aids in directing repair away from A-EJ or HR and toward NHEJ.

In a distance-dependent manner, 53BP1 mediates synapsis of distal DNA ends (Figure 5). It has been shown that 53BP1 is capable of joining breaks between 1.2 and 96 kb long, the range of repair during rearrangement in V(D)J recombination and CSR (70). This distance-dependent repair function corresponds to γ H2AX spreading and is RNF8/RNF168-dependent. 53BP1 also has been shown to associate with motor proteins, possibly to enhance chromatin mobility during repair of distal DNA breaks (70, 71). In a distance-independent manner, 53BP1 is important for DNA end protection (71, 72). 53BP1 has been shown to block access of DNA nucleases to DNA ends, possibly due to its ability to constitutively interact with H4K20me2 (59, 70, 72). End protection is vital for V(D)J recombination and CSR as it prevents formation of ssDNA microhomologies that would permit repair by A-EJ or HR repair pathways. In this way, 53BP1 directs the cell to repair DSBs by NHEJ, shunning A-EJ or HR, and foci formation is dependent upon RNF168 ubiquitin ligase activity (Figure 5).

## Concluding Remarks

While programed DNA breaks in Ig loci during B-cell development highlights the diverse repertoire of post-translation ubiquitin modifications, ubiquitination processes conversely have shed light on the vital aspects of protein regulation. Though normally avoided, DNA breaks are essential during B-cell development and for a functional adaptive immune system. Regulating the proteins and repair pathways involved during these processes is therefore of utmost importance. RAG and AID off-target activity can be detrimental to the cell, introducing deleterious mutations and/or translocations with oncogenes. E3 ligases and ubiquitination events have been shown in the context of RAG regulation, AID activity and targeting, and recruitment of NHEJ repair proteins. Ubiquitination of RAG and AID are proposed to be important in restricting DNA damage activity at undesirable loci or during incorrect cell-cycle phase. This demonstrates roles for ubiquitin beyond canonical protein degradation. As ubiquitination is involved in protein stabilization and accumulation, recruitment, and co-factor binding, the cell is presented with another layer of regulation, the ubiquitin code.

## Conflict of Interest Statement

The authors declare that the research was conducted in the absence of any commercial or financial relationships that could be construed as a potential conflict of interest.
